# The direct effect of Focal Adhesion Kinase (FAK), dominant-negative FAK, FAK-CD and FAK siRNA on gene expression and human MCF-7 breast cancer cell tumorigenesis

**DOI:** 10.1186/1471-2407-9-280

**Published:** 2009-08-12

**Authors:** Vita M Golubovskaya, Min Zheng, Li Zhang, Jian-Liang Li, William G Cance

**Affiliations:** 1 Department of Surgical Oncology, Roswell Park Cancer Institute, Buffalo, NY, USA; 2 UF Shands Cancer Center, Gainesville, FL, USA; 3 Interdisciplinary Center for Biotechnology Research, Gainesville, FL, USA

## Abstract

**Background:**

Focal adhesion kinase (FAK) is a non-receptor tyrosine kinase that plays an important role in survival signaling. FAK has been shown to be overexpressed in breast cancer tumors at early stages of tumorigenesis.

**Methods:**

To study the direct effect of FAK on breast tumorigenesis, we developed Tet-ON (tetracycline-inducible) system of MCF-7 breast cancer cells stably transfected with FAK or dominant-negative, C-terminal domain of FAK (FAK-CD), and also FAKsiRNA with silenced FAK MCF-7 stable cell line. Increased expression of FAK in isogenic Tet-inducible MCF-7 cells caused increased cell growth, adhesion and soft agar colony formation *in vitro*, while expression of dominant-negative FAK inhibitor caused inhibition of these cellular processes. To study the role of induced FAK and FAK-CD *in vivo*, we inoculated these Tet-inducible cells in nude mice to generate tumors in the presence or absence of doxycycline in the drinking water. FAKsiRNA-MCF-7 cells were also injected into nude mice to generate xenograft tumors.

**Results:**

Induction of FAK resulted in significant increased tumorigenesis, while induced FAK-CD resulted in decreased tumorigenesis. Taq Man Low Density Array assay demonstrated specific induction of FAKmRNA in MCF-7-Tet-ON-FAK cells. DMP1, encoding cyclin D binding myb-like protein 1 was one of the genes specifically affected by Tet-inducible FAK or FAK-CD in breast xenograft tumors. In addition, silencing of FAK in MCF-7 cells with FAK siRNA caused increased cell rounding, decreased cell viability *in vitro *and inhibited tumorigenesis *in vivo*. Importantly, Affymetrix microarray gene profiling analysis using Human Genome U133A GeneChips revealed >4300 genes, known to be involved in apoptosis, cell cycle, and adhesion that were significantly down- or up-regulated (p < 0.05) by FAKsiRNA.

**Conclusion:**

Thus, these data for the first time demonstrate the direct effect of FAK expression and function on MCF-7 breast cancer tumorigenesis *in vivo *and reveal specific expression of genes affected by silencing of FAK.

## Background

Focal adhesion kinase (FAK) is a 125 kDa non-receptor tyrosine kinase localized at the focal adhesions [1], which are the contact points between cells and extracellular matrix and are the sites of intense tyrosine phosphorylation [2]. FAK is tyrosine phosphorylated in response to a number of stimuli, including clustering of integrins [3], plating on fibronectin or collagen [4,5], and in response to a number of mitogenic agents [6]. FAK is involved in regulation of different cellular processes, such as cell spreading, adhesion, motility, proliferation, and survival [7]. Although several studies supported that FAK plays a role in breast carcinogenesis [8-11], the direct and specific role of FAK up and down-regulation on breast cancer tumorigenesis *in vivo *and genes expression profiling effected by FAK silencing are not understood.

FAK was originally identified as a major tyrosine phosphorylated protein in cells transformed by v-Src and associated with c-Src [12,13]. FAK is overexpressed in invasive and metastatic tumors [14], and the FAK gene is also amplified in many types of tumors [15], suggesting a role for FAK in adhesion or survival in tumor cells. In cancer cells, attenuation of FAK expression induces detachment and apoptosis [16], suggesting that a FAK-dependent signal is required for tumor cell growth. Furthermore, an activated form of FAK leads to resistance to anoikis [17], and FAK degradation is associated with apoptosis [18,19]. The C-terminal domain of FAK, called FAK-CD is analogous to murine FAK-related non-kinase (FRNK) [16], and has been shown to cause increased cell rounding, detachment, and apoptosis when transduced into breast and colon cancer cells [20-22].

Immunohistochemical analysis of FAK expression demonstrated up-regulation of FAK in 88% of invasive and metastatic breast tumors [23]. The up-regulation of FAK occurred at early stages of breast carcinogenesis [24], as FAK overexpression was detected ductal carcinoma *in situ *(DCIS) that precedes tumor cell invasion and metastasis [25]. FAK overexpression highly correlated with microvessel density, metastasis, and angiogenesis [26]. However, the studies on the role of FAK in breast tumorigenesis *in vivo *have been mostly limited to immunohistochemical studies of tumor biopsies. The recent study using Cre/loxP recombination system to disrupt FAK function in the mammary epithelium demonstrated that FAK is required for the transition of premalignant hyperplasia to carcinomas and their subsequent metastasis [27].

To determine the direct role of FAK in breast tumors *in vivo*, we created stable clones of human breast cancer cells overexpressing FAK or dominant-negative FAK-CD using the Tet-ON system and studied these cells in a nude xenograft model. In addition, we employed RT-PCR Low-density Array and Affymetrix analyses to reveal genes directly affected by FAK up or down-regulation. To the best of our knowledge, this is the first study of gene expression profiles affected by FAK regulation in MCF-7 breast cancer cell model.

## Methods

### Cells

MCF-7 cell line was purchased from ATCC and cultured in Eagle's minimal essential medium (EMEM) containing 10% fetal bovine serum (FBS), 10 μg/ml insulin, 1 mM sodium pyruvate, and 0.1 mM nonessential amino acids.

### Antibodies, plasmids and reagents

For Western blot analyses, the antibodies used were anti-FAK 4.47 (*Upstate Biologicals*), FAK; C-terminal antibody, C20 (*Santa Cruz*); HA-tag; P-418-Src, Y118-paxillin, paxillin; AKT; PY397-FAK antibodies were from *Biosource Inc*, and anti-β-Actin from *Sigma Inc*. DMP1 antibody was obtained from *Abcam Inc*. Hygromycin and doxycycline were obtained from *Clontech Laboratories, Inc*. Geneticin, G418 was obtained from *MP Biomedics Inc*. The Tet-ON gene expression System was obtained from *Clontech Laboratories Inc*. The Tet-ON system (*Clontech Laboratries Inc.*) contained pTet-ON vector, pTRE-2hyg and pTRE-2hyg-Luc vectors.

### Construction of FAK and FAK-CD-TRE-2 plasmids

The HA-tagged FAKcDNA fragment that was subcloned from pcDNA3 plasmid into BamHI and EcoRV sites of TRE-2-hyg plasmid. FAK-CD, FAK-C-terminal domain of FAK (677–1052 amino acids) was obtained by PCR. The PCR fragment was subcloned into NheI and EcoRV sites of TRE-2 plasmid. The FAK and FAK-TRE-2 plasmid sequences were confirmed by sequencing in the ICBR facility (UF, Shands Cancer Center).

### Generation of stable human doxycycline-inducible breast cancer MCF-7 cells, overexpressing FAK or dominant-negative FAK-CD

The first step was to create stably transformed MCF-7 cells by transfecting with neomycin-resistant pTet-ON regulator plasmid, encoding rtTA protein (reverse tTA, tetracycline-controlled transactivator). The stably transformed MCF-Tet-ON clones were selected by cultivation in EMEM, containing 500 μg/ml Geneticin, G418. The Tet-ON MCF-7 cells were selected and used for transfection of FAK and FAK-CD-TRE-2-hyg plasmids with Lipofectamin 2000 (*Invitrogen Inc*) according to the manufacturer's protocol. The transfected cells with TRE-2-hyg, FAK-TRE-2-hyg and FAK-CD-TRE-2-hyg plasmids were maintained in a medium with G418 (0.2 mg/ml) and hygromycin (0.1 mg/ml). The dose- and time-dependent experiments on stably transfected Tet-ON cells showed a maximal induction of FAK and FAK-CD at 2 μg/ml doxycycline at 4 days of cell cultivation. The pooled population of cells with maximal induction of FAK and FAK-CD by 2 μg/ml doxycycline used for the study.

### Generation of MCF-7 cells stably expressing FAKsiRNA

The pRNAT-H1.4-hyg plasmid was kindly provided by Dr. K. Brown (University of Florida, Gainesville). For generation of siRNA construct, the GenScript software was used. The primers for generating the hairpin construct containing FAKsiRNA were the following (with siRNA sequences shown in bold):

FAKsiRNA#1 oligonucleotides were 5'-CGCGTCG**TAATACTCGCTCCATTGCACC**TT GATATCC**GGGTGCAATGGAGCGAGTATTA**TTTTTTCCAAC-3' and complementary oligonucleotide. For FAKsiRNA#2, oligonucleotides were the following: 5'-CGCGTCG**TA AATGCCTTGATGTACATCT**TTGATATCCG**AGATGTACATCAAGGCATTTA**TTTTT TCCAAC-3' and the complementary oligonucleotide. For control siRNA, scrambled FAK siRNA or firefly luciferase oligonucleotides were used. Oligonucleotides for control siRNA (scrambled FAK siRNA#1 sequence) were generated with GenScript software and oligonucleotides were the following: 5'-CGCGTCG**CCACTTACGATAGCTCCATTC**T TG ATATCCG**GAATGGAGCTATCGTAAGTGG**TTTTTTCCAAC-3' and the complementary oligonucleotide. Firefly luciferase siRNA was also used as a control, and the oligonucleotides were 5'-CGCGTCGTCGAAGTACTCAGCGTAAGTTGATATCCGCTTACGCTGAGTACTT CGATTTTTTCCAAC-3' and the complementary oligonucleotide. The oligonucleotide duplexes were subcloned into MluI and XhoI sites of pRNAT-H1.4-hyg plasmid. The resulting siRNA DNAs were used for transfecting cells with Lipofectamin 2000 (*Invitrogen Inc*) according to the manufacturer's protocol. The MCF-7 cells were transfected with control vector, control siRNA, FAKsiRNA#1, and FAKsiRNA#2 and after several weeks growing in the presence of hygromycin (100 μg/ml), stable clones were collected and analyzed by Western immunoblotting with FAK antibodies. The stable cell line with highest reduction of FAK expression was used for the study.

### Western Blotting

Western Blotting was performed as described before [28].

### Immunohistochemistry staining of xenograft tumors

Tumors from untreated and treated with doxycycline-treated mice were removed and fixed in 4% formaldehyde solution immediately after surgical resection. The fixed samples were analyzed in the Immunohistochemistry Core Facility (UF, Department of Pathology). FAK staining was performed, as described previously with FAK 4.47 antibodies [29].

### Cell growth *in vitro*

2 × 10^5 ^cells were plated on 6-well plates. Cell growth was determined by counting cells on hemocytometers. Trypan blue exclusion assay was used for detecting viable cells.

### RNA isolation

Total cellular RNA was isolated from cultured cells with a NucleoSpin RNA II Purification Kit (*Clontech Laboratories, Inc*.) according to the manufacturer's protocol.

### Taq Man Low Density Array Real time PCR assay

Customized Taq Man Low density arrays with 44 different genes (Table 1) and a GAPDH probe as a normalization control were obtained from *Applied Biosystems*. The isolated RNA was used for PCR reaction as described in the manufacturer's protocol. The ABI PRISM 7700 cycler's software calculated a threshold cycle number (Ct) at which each PCR amplification reached a significant threshold level. The relative quantity, RQ, was calculated and statistical analysis was performed with Student's t-test.

**Table 1:** Fold changes of mRNA expression levels in MCF-7-Tet-ON cell lines cultivated in the presence or absence of doxycycline (2 μg/ml) for 6 days

Gene Symbol	Dox^+^/Dox^- ^Ratio		
	Tre-2	FAK	FAK-CD
PTK2	0.59	1.86 *	0.82
PTK2B	0.82	0.85	0.64
p53	0.99	0.66	0.85*
DMTF1	1.20	0.65*	0.81
SRC	1.95	0.76	0.87
MAPK3	1.05	0.74	0.97
AKT1	0.56	0.58	0.73
MAP2K1	0.57	0.52*	0.52
MAPK8	0.70	0.61*	0.85*
FYN	0.84	0.33*	0.65*
CDC2	0.72	1.02	0.82*
CDK4	0.94	0.88*	0.94*
RB1	0.88	0.48*	0.60*
SOCS2	1.12	0.65*	1.20*
SYK	0.66	0.47*	0.63*
CDK2	0.97	0.93*	0.73
CDK3	0.80	0.71	0.74
RAF1	0.78	0.60*	0.54*
ABL1	0.63	0.66	0.78*
TEC	0.67	0.63*	0.58*
PXN	0.96	0.62*	0.75*
SHC1	0.98	0.62*	0.78
BCAR1	0.62	0.53	0.70
MAK2K6	0.76	1.04	1.73*
EPHA1	0.56	0.78	0.75
CTNNB1	0.80	0.56*	0.70*
CHEK1	0.75	0.62*	0.62*
ATM	1.09	0.65	0.99
BIRC5	0.93	0.88	0.74
CDC25C	1.27	1.05	1.18
BCL2	0.31	0.30	0.37
TLN2	0.72	0.81*	0.65*
FLT1	0.33	0.37	0.46
PINK1	1.18	0.51*	0.65*
STAT1	0.70	0.54*	0.46
ARHGEF2	0.75	0.56	0.73
ITGB1	0.46	0.52*	0.65*
CELSR1	1.26	0.88	0.79
LAMC2	0.64	0.38*	0.64*

*p < 0.05, t-test; ** mRNA levels of some genes from Additional file 1 was not detectable

### Microarray and statistical data analyses

RNA was isolated from MCF-7, MCF-7-Vector, MCF-7-Control luciferase siRNA, FAKsiRNA#1, and FAKsiRNA#2 samples using *Clontech Labs, Inc*. Kit. The cDNA preparation, probe labeling, hybridization and image analysis of the arrays were carried at ICBR Core Facility (UF) according to the manufacturer's recommendations (*Affymetrix*, Santa Clara, CA, USA), using Affymetrix Human Genome U133A GeneChip containing 47000 transcripts. For the data analysis, we treated siRNA#1, siRNA#2 as siRNA group, and MCF7, MCF-7-Vector and MCF-7-Control siRNA as control group. We used with 4 replicates per group in the analysis. Affymetrix Microarray software was used to analyze the data.

Statistical tests were performed using BioConductor statistical software http://www.bioconductor.org/[30]. Data pre-processing and normalization were performed using the Affy package [31]. Raw data were normalized by Robust Multichip Analysis (RMA) approach. The local pooled error (LPE) method [32] was applied to detect the genes which are significant differentially expressed between FAK siRNA and control (MCF-7-Vector and Control siRNA) samples, and the resampling technique was used to control the false-discovery rate (FDR). In combination with a resampling FDR correction, the LPE method was shown to outperform other 2-sample comparison methods [32]. We used local-pooled-error (LPE) approach to evaluate the level of significance for each gene's differential expression. The LPE estimation is based on pooling errors within genes and between replicate arrays for genes in which expression values are similar. The p-values from LPE were used as first criteria to define the significant gene set. The complete data are uploaded to NCBI website, Accession number GSE11581. Differentially expressed genes were ranked by the p-values, and the genes with p < 0.05 were considered as differentially expressed genes at a statistically significant level. For those significant genes, different level of fold-change were used to select the significant differentially expressed genes. Cluster analysis was performed on these selected differentially expressed genes using hierarchical clustering with the complete linkage method on a similarity matrix built with Pearson correlation coefficient. The results of the cluster analysis were displayed at heat map. The data are deposited in NCBI database (NCBI Accession number GSE11581).

### Soft agar growth assay

Cells cultivated for 6 days with or without doxycycline (2 μg/ml) were plated in 0.3% agar (with or without doxycycline) on the plates, containing 0.5% agar (with or without doxycycline). The plates were cultivated on the plates for 2–3 weeks. The colonies were counted on stained with crystal violet plates under the microscope. Samples were assayed in duplicates.

### Adhesion Assay

96-well plates coated with collagen were blocked in 0.5% BSA in the EMEM medium. Then 2 × 10^4 ^of cells were plated on collagen-treated plates and incubated at 37°C for 37 minutes in CO_2 _incubator. Cells were washed with PBS and fixed with 3.7% formaldehyde for 10 minutes. After washing with PBS, cells were stained with crystal violet (5 mg/ml in 2% ethanol). Then 2% SDS was added and plate was read at 590 nm to detect adhesion of cells.

### Tumor growth in nude mice

Female nude mice (4–5 weeks old) were ordered from *Harlan Laboratories Inc*. All experiments were performed according to guidelines of the approved IACUC protocol. To supplement the estrogens for MCF-7 proliferation each nude mouse was implanted with a 1.5 mg of 17β-estradiol pellet (*Innovative Research of America, Sarasota, FL*, *USA*). A week after the pellet implantation, 5 × 10^6 ^of MCF-7-Tet-ON cells stably expressing FAK-TRE-2 or FAK-CD were subcutaneously injected into the mice. Mice were divided into two groups: the first group did not receive doxycycline in the drinking water, and the second group received doxycycline (2 mg/ml) in the water. For FAKsiRNA experiments, 5 × 10^6 ^of MCF-7 cells were injected subcutaneously into mice with implanted 1.5 mg of 17β-estradiol pellet and tumor growth was observed. The tumors were measured using calipers and the tumor volume was calculated using the formula volume, V = L × W^2^/2, where L-long diameter and W-short diameter.

### Statistical analyses

Student's t test was performed to determine significance. The difference between data with p < 0.05 was considered significant.

## Results

### Doxycycline induces expression of FAK and FAK-CD in MCF-7-Tet-ON breast cancer cells

**Figure 1:**
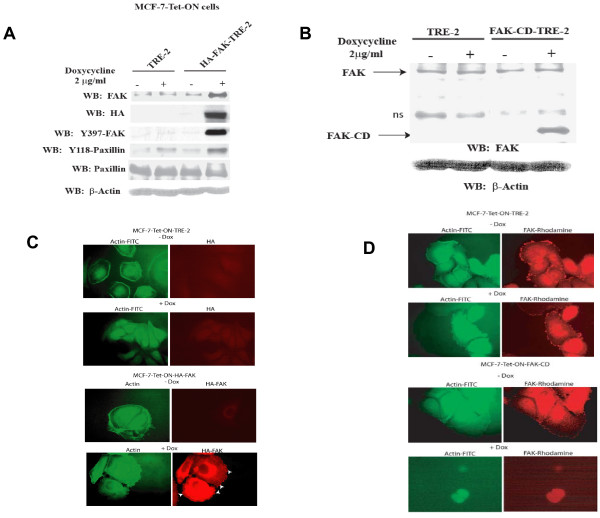
**Doxycycline induces FAK and FAK-CD in MCF-7-Tet-ON cell line**. A, B, MCF-7-Tet-ON stable cell line was generated with stable expression of FAK (A) or FAK-CD (B). Addition of doxycycline (2 μg/ml) for 6 days resulted in overexpression of FAK or FAK-CD. Western blotting with HA-tag antibody was performed to detect HA-tagged FAK. Western blotting with Y397 antibody shows expression of activated FAK in MCF-7-Tet-ON-FAK cells. Western blotting with 118-Y-paxillin antibody demonstrates increased phosphorylation of FAK-substrate paxillin in MCF-7-Tet-ON-FAK cells. Western blotting with beta-actin shows equal loading of proteins. C, Exogenous FAK localizes to focal adhesions. MCF-7-Tet-ON-HA-FAK or control MCF-7-Tet-TRE-2 cells. Immunostaining with HA-tag antibody was performed to detect HA-tagged FAK. Staining with FITC-conjugated phalloidin detected actin in the cells. Dox, doxycyclin. D, Cell rounding and displacement of FAK from the focal adhesions in MCF-7-Tet-ON-FAK-CD cells. To detect FAK in the cells, we stained cells for FAK with 4.47 N-terminal FAK antibody. Upper panels: MCF-7-Tet-ON-TRE-2 cells, lower panels: MCF-7-Tet-ON-FAKCD cells.

To understand the biologic role of FAK in breast cancer cells, stable clones overexpressing FAK with a HA-tag were created from MCF-7 breast cancer cells using the Tet-ON system. The MCF-7-Tet-ON cells stably expressing FAK or FAK-CD were cultivated in the presence of doxycycline and Western blotting was performed with FAK antibodies (Figure 1). Expression of FAK (Figure 1A) and FAK-CD (Figure 1B) was induced by doxycycline compared to the cells without doxycycline. Western blotting analysis with HA-tag antibody confirmed expression of HA-tagged FAK (Figure 1A). Doxycycline-induced FAK was activated, resulting in increased Y397 autophosphorylated FAK and increased phosphorylation of FAK substrate, Y118-paxillin (Figure 1A).

To determine whether exogenous HA-tagged FAK localized to focal adhesions, immunostaining with HA-tag antibody was performed. Doxycycline-inducible FAK mainly localized to the cytoplasm, perinuclear area and the focal adhesions in MCF-7-Tet-ON-FAK cells (Fig. 1C, lower panels), while control MCF-7-Tet-ON-TRE-2 cells were negative by immunostaining with HA-tag antibody (Figure 1C, upper panels). Induction of FAK with doxycycline did not change stress fiber formation, compared to the cells without doxycycline, as detected by staining actin with phalloidin (Figure 1C).

**Figure 2:**
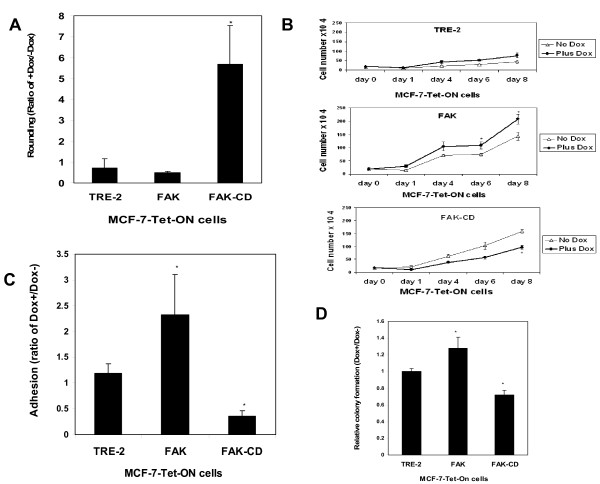
**The effect of induced FAK and FAK-CD in MCF-7-Tet-ON cell line on cell growth, rounding, adhesion and soft agar colony formation *in vitro***. A, FAK-CD induction with doxycycline causes increased rounding of MCF-7-Tet-ON cells. The ratio of rounded cells cultivated either with doxycycline 2 μg/ml for 6 days or without doxycycline treatment (Dox^+^/Dox^- ^ratio) is shown. Bars show average of three independent field counts ± standard deviations. *, p < 0.05, Student's t-test. B, Cells with doxycycline-induced FAK had increased cell growth *in vitro*, while cells with doxycycline-induced FAK-CD expressed decreased cell growth. MCF-7-Tet-ON-TRE-2 control, -FAK or -FAK-CD cells were grown without and with doxycycline for 1–8 days and the cell number of viable cells detected with trypan blue dye is plotted on the graph. *, p < 0.05, Student's t-test. Bars show average ± standard deviations of three independent experiments. C, Cells with doxycycline-induced FAK expressed increased cell adhesion on collagen, while cells with induced FAK-CD had decreased adhesion. The doxycycline-induced adhesion is expressed relative to the adhesion of cells without doxycycline. Bars show average ± standard deviations of three independent experiments. *, p < 0.05, Student's t-test. D, Cells with doxycycline-induced FAK had increased soft agar colony formation, while cells with FAK-CD overexpression had decreased colony formation. The number of soft agar colonies on plates with doxycycline is expressed relative to the number of colonies on plates without doxycycline *, p < 0.05, Student's t-test.

To detect the effect of exogenous FAK-CD on FAK localization in cells with doxycycline-inducible FAK-CD overexpression, we stained FAK with the N-terminal domain antibody (Figure 1D). Doxycycline caused displacement of FAK from the focal adhesion and induced cell rounding (Figure 1D). To quantify the cell rounding, we counted the percent of rounded cells caused by doxycycline-inducible FAK-CD (Figure 2A). Rounding in the presence of doxycycline was induced more than 5 times in the MCF-7 cells overexpressing FAK-CD. In contrast, there was no cell rounding caused by overexpression of FAK compared to the TRE-2 stable control cells (Figure 2A). Thus, doxycycline-induced FAK-CD caused changes in cell morphology resulting in cell rounding.

### Doxycycline-inducible FAK increases MCF-7 cell growth, adhesion and soft agar colony formation, while FAK-CD decreases these cellular processes

MCF-7 cells expressing TRE-2, FAK-TRE-2 or FAK-CD-TRE-2 were grown without and with doxycycline for 8 days, and viable cells were counted (Figure 2B). Control MCF-7-Tet-ON-TRE-2 cells that did not significantly change cell growth *in vitro *(Figure 2B). In contrast, cells with doxycycline-inducible FAK expressed significantly (p < 0.05) increased cell growth at 6–8 days, while cells with induced FAK-CD had significantly (p < 0.05) decreased cell growth (Figure 2B). To detect the effect of overexpressed FAK and FAK-CD on cell adhesion, we plated MCF-7 cells on collagen and performed adhesion assay (Figure 2C). Doxycycline-inducible FAK resulted in induction of cell adhesion compared to the cells without doxycycline, while FAK-CD induction resulted in significantly decreased cell adhesion (Figure 2C).

To detect whether FAK and FAK-CD affected anchorage-independent growth, cells were treated with doxycycline (2 μg/ml) for 6 days, resuspended in 0.3% agar, with or without doxycycline, and cells were cultivated for 3 weeks on soft agar, and colonies were counted. Doxycycline-induced FAK resulted in an increased number of colonies compared to cells without doxycycline, while FAK-CD resulted in decreased colony formation (Figure 2D).

Thus, overexpression of FAK increases breast cancer cell growth, adhesion and soft agar colony formation *in vitro*, while dominant-negative FAK, FAK-CD decreases these cellular processes.

### Doxycycline-inducible FAK induces tumor growth, while doxycycline-inducible FAK-CD reduces the tumor growth in nude mice *in vivo*

**Figure 3:**
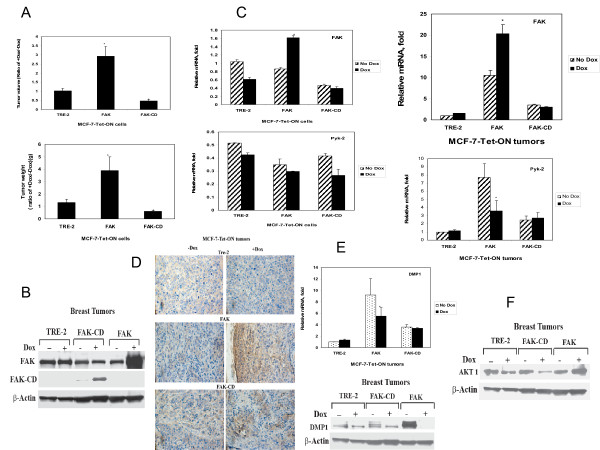
**Doxycycline-induced FAK increases tumorigenesis in xenograft mice model *in vivo *in contrast to FAK-CD**. A, MCF-7-Tet-ON-TRE-2 control cell line, MCF-7-Tet-ON-FAK and MCF-7-Tet-ON-FAK-CD cells were injected into the nude mice. Mice were divided on two groups (n = 5–7) that were given either drinking water without or with doxycycline. Doxycycline significantly increased volume and weight, in mice with injected MCF-7-Tet-ON-FAK cells in contrast to FAK-CD. Bars show means ± standard errors. *, p < 0.05, Student's t-test. B, Doxycycline induced FAK expression in xenograft tumors with injected MCF-7-Tet-ON-FAK cells and induced FAK-CD expression in case of MCF-7-Tet-ON-FAK-CD. Western blotting was performed on tumor lysates with FAK-C20, C-terminal antibody for detecting FAK and FAK-CD. C, TaqMan RT-PCR analysis of gene expression in MCF-7-Tet-ON cells and in MCF-7-Tet-ON xenograft tumors. Real-time PCR analysis was performed on breast cancer cell lines MCF-7-Tet-ON-TRE-2, -FAK or -FAK-CD grown for 6 days either without or with doxycyclin (2 μg/ml) (left panel) and on tumor samples from these injected cell lines without or with doxycycline (right panel) (Materials and Methods). Expression of genes was calculated relative to GAPDH control (RQ) and normalized to MCF-7 cells in case of cell lines and relatively to MCF-7-Tet-ON-TRE-2 without doxycycline in case of tumor samples. Bars show means ± standard errors of two independent experiments. *p < 0.05, FAK mRNA level in MCF-7-Tet-ON-FAK (+Dox) versus MCF-7-Tet-ON-FAK(-Dox) samples Student's t-test. Left upper panel: FAK expression in MCF-7-Tet-ON breast cell lines, Right upper panel: FAK expression in tumor samples (two tumors for each group was used without doxycycline and with doxycycline). Left lower panel: Pyk-2 expression in MCF-7-Tet-ON breast cell lines. Right lower panel: Pyk-2 expression in tumor samples (two tumors for each group was used without doxycycline and with doxycycline). D, Immunohistochemical analysis of MCF-7-Tet-ON tumors. MCF-7-Tet-ON-FAK tumors demonstrate increased FAK expression in the presence of doxycycline. E, Decreased expression of DMP1 gene in MCF-7-Tet-ON-FAK tumors in the presence of doxycycline. Upper panel: The Real-Time PCR analysis by TaqMan array assay on tumor samples. Bars show average ± standard errors of two independent experiments. * p < 0.05, FAK +Dox tumors versus FAK-Dox tumors, Student's t-test. Lower panel: Western with DMP1 antibody was performed on tumor cell lysates. DMP1 protein level was significantly lower in MCF-7-Tet-ON-FAK tumors in the presence of doxycycline compared with MCF-7-Tet-ON-FAK tumors without doxycycline. F, Decreased expression of AKT1 in MCF-7-Tet-ON-FAK-CD tumors in the presence of doxyxycline. Western blotting with AKT1 antibody was performed on tumor cell lysates, as described above. AKT 1 expression is significantly decreased in MCF-7-Tet-ON-FAK-CD tumors in the presence of doxycycline. * p < 0.05, FAK-CD Dox^+ ^tumors versus FAK-CD Dox^- ^tumors, Student's t-test.

To determine role of FAK and FAK-CD in breast tumorigenesis, we injected MCF-7-Tet-ON-FAK, FAK-CD and TRE-2-vector stable cell lines into two groups of nude mice given drinking water either with or without doxycycline and observed tumor growth. Doxycycline-induced FAK significantly increased tumor volume, more than 2.9 times that of control TRE-2 mice (Figure 3A, upper panel). Induction of FAK-CD had a 3-fold decreased tumor volume (Figure 3A, upper panel). A similar result was observed with tumor weights (Figure 3A, lower panel). Western blotting with FAK-C-terminal antibody showed an increased level of FAK and FAK-CD in tumors of doxycycline-treated mice with induced FAK and FAK-CD expression, respectively (Figure 3B). Thus, induced FAK expression increased breast tumor growth in mice xenograft model and FAK-CD decreased tumor growth *in vivo*.

### TaqMan Low Density Array analysis in Tet-inducible MCF-7-Tet-ON-FAK cell lines and tumors demonstrates specific overexpression of FAK

To determine the molecular mechanism of FAK and FAK-CD induction, we performed TaqMan low-density array analysis in Tet-inducible MCF-7 cell lines. We compared the expression of genes inside each isogenic cell line grown with and without doxycycline. We compared expression of 44 genes relative to GAPDH control in all cell lines (Additional file 1). The expression levels of expression of 39 genes with detectable mRNA levels that were differentially affected by Tet-inducible FAK or FAK-CD-induction are shown in Table 1. Real-time PCR demonstrated an increased 1.9 fold expression of FAK in MCF-Tet-ON-FAK cells cultivated in the presence of doxycycline (2 μg/ml) for 6 days (Figure 3C, upper left panel), while control MCF-7-Tet-ON-TRE-2 and MCF-7-Tet-ON-FAK-CD cells did not demonstrate increased expression of FAK mRNA in the presence of doxycycline (Figure 3C, upper left panel). Expression of the FAK homologue Pyk-2 was not significantly changed in any of the cell lines in the presence of doxycycline (Figure 3C, lower left panel). The RNA level of other genes was not significantly affected by induction of FAK compared with MCF-7-Tet-ON-TRE-2 and MCF-7-Tet-ON-FAK-CD cells. Interestingly, we found that cyclin D binding myb-like protein 1 (DMP1; also called DMTF1) was 1.5 fold decreased (p < 0.05) in MCF-7-Tet-ON-FAK cells in the presence of doxycycline, and was not affected in MCF-7-Tet-ON-TRE-2 or MCF-7-Tet-ON-FAK-CD-expressing cell lines. Thus, increased expression of FAK is consistent with increased tumorigenesis in MCF-7-Tet-ON xenograft cells in the presence of doxycycline.

To determine that tumors will have increased expression of FAK in the case of an induced MCF-7-Tet-FAK model, we performed the TaqMan Low Density Array PCR analysis in tumors and confirmed increased expression of FAK in MCF-7-Tet-ON xenografted tumors (Figure 3C, upper right panel). Tumors with doxycycline-inducible expression of FAK had significantly increased levels of FAK mRNA compared with tumors of mice without doxycycline induction and compared with TRE-2 control or tumors with FAK-CD-induction (Figure 3C, right panel). Expression of FAK homolog Pyk-2 was not increased and even was decreased in the MCF-7-FAK tumors in the presence of doxycycline, and it did not change significantly in TRE-2 and FAK-CD group in the presence of doxycycline (Figure 3C, lower right panel). Increased expression of FAK mRNA observed in the presence of doxycycline in Tet-ON-FAK-tumors was consistent with increased FAK protein level demonstrated by Western blotting (Figure 3B) and by immunohistochemical staining (Figure 3D).

Among the genes, specifically affected by FAK or FAK-CD overexpression in the presence of doxycyclin, we found that the DMP1 mRNA expression was > 1.7 fold decreased (p < 0.05) in MCF-7-Tet-ON-FAK tumors grown in the presence of doxycycline, and the difference was not significant in case of TRE-2-control or FAK-CD tumors (Figure 3E, upper panel). Decreased expression of DMP1 protein in MCF-7-Tet-ON-FAK tumors grown in the presence of doxycycline was confirmed by Western blotting (Figure 3E, lower panel). In the case of MCF-7-Tet-ON-FAK-CD tumors that were grown in the presence of doxycycline, the level of AKT1 mRNA decreased >1.3 fold (p < 0.05), while the difference was not significant in MCF-7-Tet-ON-TRE-2 or MCF-7-Tet-ON-FAK tumors The same result was obtained by Western blotting with AKT antibody (Figure 3F). The AKT1 expression was decreased in MCF-7-Tet-ON-FAK-CD tumors that were grown in the presence of doxycycline (Figure 3F).

Thus, increased tumorigenesis was observed in the tumors with MCF-7-Tet-ON-FAK cells and decreased tumorigenesis was observed in the case of expression dominant-negative FAK inhibitor FAK-CD.

### FAK siRNA causes cell rounding, decreases cell growth *in vitro *and inhibits tumorigenesis *in vivo*

**Figure 4:**
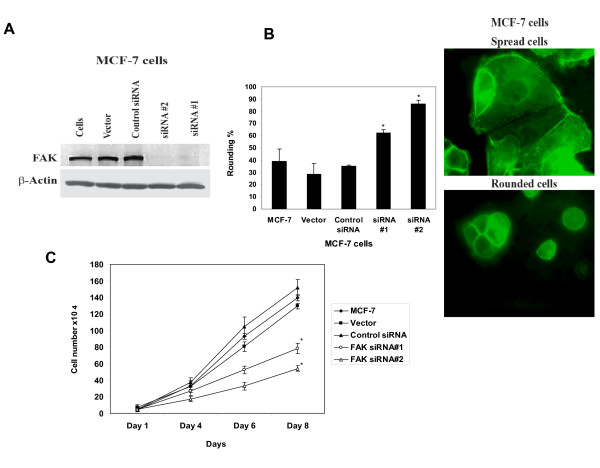
**FAKsiRNA significantly decreased FAK and caused MCF-7 cell rounding and decreased cell growth *in vitro *and tumorigenesis *in vivo***. A, Western blotting demonstrates that FAKsiRNA#1 and FAKsiRNA #2 decreased expression of FAK in MCF-7 cells, while Control siRNA and Vector control samples did not affect FAK expression. B, FAKsiRNA causes increased cell rounding. GFP-positive MCF-7, MCF-7-Vector, MCF-7-Control siRNA and FAKsiRNA#1 and #2 cell lines with rounded morphology (shown on right panels) were counted on three separate fields with 100 cells counted per field. The average percent of rounding is shown ± standard errors. *, p < 0.05, FAKsiRNA versus control samples. C, FAKsiRNA caused decreased cell growth *in vitro*. MCF-7, MCF-7-Vector, MCF-7-Control (luciferase) siRNA and FAKsiRNA#1 and #2 cells were plated on a 6-well plate, cultivated for 1, 4, 6 and 8 days were and counted on hemocytometer with trypan blue for detection of viable cell growth. FAKsiRNA#1 and #2 significantly decreased cell growth compared to control MCF-7, MCF-7-Vector or Control siRNA cells. *, p < 0.05 FAKsiRNA#1 and FAKsiRNA#2 versus control cells.

To test the effect of decreased FAK expression and compare with the FAK-CD-induced expression, we generated stable MCF-7 cell lines, expressing vector alone, Control siRNA, FAKsiRNA#1 and FAKsiRNA#2. All stably transfected cells were GFP-positive showing effective expression of protein, since vector had GFP-tag. Western blotting with FAK antibody demonstrates that both FAKsiRNA #1 and #2 cells efficiently decreased expression of FAK in MCF-7 cells, while MCF-7, MCF-7-Vector or MCF-7-Control siRNA did not decrease FAK expression (Figure 4A). FAKsiRNA caused change in cell morphology and significantly increased cell rounding compared with MCF-Vector or Control siRNA (Figure 4B). In addition, FAKsiRNA significantly decreased cell growth *in vitro *compared with MCF-7, Vector and Control siRNA cells (Figure 4C). Thus, FAK siRNA caused cell rounding and decreased cell growth *in vitro*.

**Figure 5:**
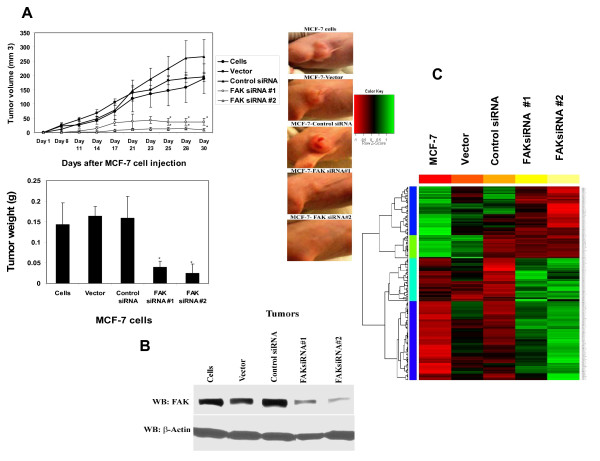
**Decreased tumorigenesis *in vivo *and gene expression analysis in MCF-7-FAKsiRNA model**. A, Upper left panel, MCF-7, MCF-7-Vector, MCF-7-Control siRNA (firefly luciferase siRNA), MCF-7-FAKsiRNA#1 and FAKsiRNA#2 were injected into mice, and tumor volume was measured at different time points (1–30 days). FAKsiRNA #1 and #2 decreased tumor growth *in vivo *compared to MCF-7, MCF-7-Vector and MCF-7-Control siRNA cells. Bars show means of tumor growth measured at day 30 ± standard errors (n = 5 mice in each group). Lower left panel: Tumors from FAKsiRNA cells had significantly less weight than tumors from control MCF-7, MCF-7-Vector and MCF-7-Control siRNA group (measured at day 30). *, p < 0.05 FAKsiRNA#1 and FAKsiRNA#2 versus control cells. Right panel: Representative tumor sizes are shown from each group. B, Tumors collected at day 30 from MCF-7-FAKsiRNA mice express decreased levels of FAK. Western blotting was performed with FAK antibody. C, Profile of gene expression in FAKsiRNA cell lines with Affymetrix gene Chips. Microarray heat map of genes on MCF-7 cell lines is shown. Upregulated genes (green) and down-regulated (Red). Affymetrix gene Chip Human Genome U133A (two chips per sample) was used. More than 4300 genes (shown) up or down-regulated with p < 0.05 in FAKsiRNA#1 and FAKsiRNA#2 pooled group compared to MCF-7, MCF-7-Vector and MCF-7-Control siRNA pooled group.

To test the effect of down-regulated FAK on tumorigenesis, we injected MCF-7, MCF-7-Vector, MCF-7-Control siRNA, MCF-7-FAKsiRNA and FAKsiRNA#2 cells in the nude mice and observed tumor growth. Both FAKsiRNA #1 and #2 decreased tumor cell growth compared to MCF-7 and MCF-7-Vector and MCF-7-Control siRNA cells (Figure 5A, left upper panel). The significantly decreased tumor size in case of FAK siRNA#1 and #2 is shown on Figure 5A, right panels. Both FAK siRNA #1 and #2 significantly decreased tumor volume versus control MCF-7, MCF-7-Vector and MCF-7-Control siRNA cells (Figure 5A, left lower panel). We performed analysis of FAK expression in these tumor samples by Western blotting (Figure 5B). Tumors from both FAKsiRNA#1 and FAKsiRNA#2 had significantly decreased FAK expression compared with tumors from MCF-7, MCF-7-Vector and MCF-7-Control siRNA group (Figure 5B). Thus, down-regulation of FAK by FAK siRNA significantly decreased tumor growth *in vivo *compared to MCF-7 and MCF-7-Vector and MCF-7-Control siRNA cell lines.

### Affymetrix Microarray and TaqMan Low-Density RT-PCR array assays reveal more than 4300 genes down- and up-regulated in MCF-7-FAKsiRNA cell lines

In addition, to better understand molecular mechanism of FAK down-regulation by FAKsiRNA, we performed gene expression of MCF-7, MCF-7-Vector, MCF-7-Control siRNA, FAKsiRNA#1 and FAKsiRNA#2 cell line samples by microarray analysis using Affymetrix Human Genome U133 GeneChip Array, covering the whole human genome with >47000 transcripts. Among all genes on the Chips, we revealed over than 4300 genes (all gene profiles, affected by FAKsiRNA are downloaded to NCBI databases with [Accession number GSE11581]) were significantly with p < 0.05 either up- or down-regulated in FAKsiRNA#1 and FAKsiRNA#2 cell lines versus control group (MCF-7, MCF-7-Vector, MCF-7-Control siRNA cell lines). The heatmap of 4300 genes that were significantly down-regulated and up-regulated (p < 0.05) by FAKsiRNA in MCF-7 cells is shown on Figure 5C.

169 genes were more than 2-fold down or up-regulated (p < 0.05), 32 genes were more than 3-fold down- or up-regulated (p < 0.05), and 12 genes were > 4-fold (p < 0.05) affected by FAKsiRNA. We found that expression of FAK was 1.5-fold down-regulated (p < 0.05) in the pooled FAKsiRNA#1 and FAKsiRNA#2 group compared to control group (Table 2). TaqMan Real-time PCR array assay demonstrated the same 1.5 fold decrease of FAKmRNA by both FAKsiRNA compared to control MCF-7 and MCF-7-Vector cells, confirming and validating microarray data. Importantly, Pyk2 and several other genes were not significantly different by both microarray and Real-time-PCR, additionally validating both methods.

**Table 2:** Several sets of Up-regulated and Down-regulated genes (p < 0.05) after FAK silencing with FAKsiRNA*

	Down-regulated genes			
**Probeset ID**	**Gene Symbol**	**Gene name**	**Gene Ontology (GO) Biological process**	**Fold change siRNA/Control**

212094_at	PEG10	Paternally expressed 10	Proteolysis; apoptosis; differentiation	0.22
205239_at	AREG	Amphiregulin	Cell-cell signaling; proliferation	0.23
223062_s_at	PSAT1	Phosphoserine Aminotransferase 1	L-serine biosynthetic; metabolic; amino-acid biosynthetic processes	0.29
214467_at	GPR65	G protein-coupled receptor 65	Apoptosis; G-protein coupled receptor protein signaling pathway; immune response	0.31
238695_s_at	RAB39B	RAB39B, member RAS oncogene family	Transport; small GTPase mediated signal transduction; protein transport	0.39
239014_at	CCAR1	Cell division cycle and apoptosis regulator 1	Apoptosis; cell cycle; cell division	0.42
231879_at	COL12A1	Collagen, type XII, alpha 1	Skeletal development; phosphate transport; cell adhesion; collagen fibril organization	0.44
203424_s_at	IGFBP5	Insulin-like growth factor binding protein 5	Regulation of cell growth; signal transduction	0.49
235412_at	ARHGEF7	Rho guanine nucleotide exchange factor (GEF)7	Regulation of Rho protein signal transduction; intracellular signaling cascade	0.52
216765_at	MAP2K5	Mitogen-activated protein kinase kinase 5	Protein amino acid phosphorylation; signal transduction	0.61
223746_at	STK4	Serine/threonine kinase 4	Cell morphogenesis; protein amino acid phosphorylation; apoptosis kinase cascade	0.65
1559529_at	PTK2	PTK2 protein tyrosine kinase 2	Protein amino acid phosphorylation; integrin-mediated signaling pathway; signal complex assembly	0.68
205397_x_at	SMAD3	SMAD family member 3	SMAD protein complex assembly; regulation of transcription from RNA polymerase II promoter	0.70
	**Up-regulated genes**			
204214_s_at	RAB32	RAB32, member RAS oncogene family	Small GTPase mediated signal transduction; Protein transport	1.52
204794_at	DUSP2	Dual specificity phosphatase 2	Inactivation of MAPK activity; Protein amino acid dephosphorylation	1.86
206898_at	CDH19	Cadherin 19, type 2	Cell-cell adhesion; cell adhesion	2.42
201117_s_at	CPE	Carboxypeptidase E	Neuropeptide signaling pathway; Insulin processing; metabolic process	2.64
205749_at	CYP1A1	Cytochrome P450, family 1, subfamily A, polypeptide 1	Dibenzo-p-dioxin metabolic process; oxidation reduction	2.97
201010_s_at	TXNIP	Thioredoxin interacting protein	Transcription; cell cycle; keratinocyte differentiation	3.26
232306_at	CDH26	Cadherin-like 26	Cell adhesion	4.05
205792_at	WISP2	WNT1 inducible signaling pathway protein 2	Cell growth; adhesion; signal transduction	4.09
207173_x_at	CDH11	Cadherin 11, type 2, OB-cadherin (osteoblast)	Skeletal development; adhesion; ossification	4.12
204653_at	TFAP2A	Transcription factor AP-2 alpha	Transcription; signal transduction	4.99
228697_at	HINT3	Histidine triad nucleotide binding protein 3	Signal transduction	5.33
229700_at	ZNF738	Zinc finger protein 738	Regulation of transcription	8.7
205051_s_at	KIT	v-kit Hardy-Zuckerman 4 feline sarcoma viral oncogene homolog	Protein amino acid phosphorylation; signal Transduction; epithelial proliferation	12.59

* The complete set of genes down-regulated or up-regulated by FAKsiRNA with p < 0.05 are up-loaded to NCBI database (NCBI Accession number GSE11581).

Among genes that were significantly (p < 0.05) down-regulated by both FAKsiRNA#1 and FAKsiRNA#2 versus control (MCF-7, MCF-7-Vector and MCF-7-Control siRNA) group were PEG10, Paternally expressed 10 gene; AREG, Amphiregulin; insulin-like growth factor binding protein 5; MAP2K5, Mitogen-activated protein kinase 5 (Table 2). Among genes significantly up-regulated (p < 0.05) by both FAKsiRNA#1 and #2 versus control group were TXNIP, thioredoxin interacting protein (>3.2 fold); TFAP2A, transcription factor AP-2 (5 fold) (Table 2). Several of the genes were > 4–6 times either down-regulated (such as WISP2, CDH11) or up-regulated (TFAP2A, HINT3, ZNF738 and KIT) (p < 0.05) by FAKsiRNA. (Table 2). In addition, we performed Affymetrix analysis on MCF-7-Tet-ON-FAK cells, cultivated either with doxycycline (2 μg/ml) for 6 days or without doxycycline. We revealed 510 genes that are up- or down-regulated in MCF-7-Tet-ON-FAK with induced FAK (p < 0.05) (all gene profiles, affected by FAK-induction in MCF-7-Tet-ON-FAK cells are downloaded to NCBI databases with [Accession number GSE11581]). There were several genes that were inversely regulated in MCF-7-Tet-ON-FAK cells, cultivated either in the presence of doxycycline (2 μg/ml) or without doxycycline, and in MCF-7-FAKsiRNA cells. Some genes were down-regulated in MCF-7-Tet-ON-FAK cells with induced FAK and increased in MCF-7 cells with silenced FAK, such as TXNIP that was 2.7-fold (p < 0.05) decreased in MCF-7-Tet-ON-FAK cells (Dox^+^/Dox^- ^ratio = 0.36) compared to increased in FAKsiRNA cells (Table 2); IFI27 (interferon, alpha-inducible protein 27) was significantly decreased in MCF-7-Tet-ON-FAK cells (Dox^+^/Dox^- ^ratio = 0.6) compared to 2.2-fold induction by FAKsiRNA cells; CD36 (CD36 molecule (thrombospondin receptor)) was significantly down-regulated in MCF-7-Tet-ON cells (Dox^+^/Dox^- ^ratio = 0.360, while it was 2.4-fold up-regulated in MCF-7-FAKsiRNA cells. In contrast, other genes, such as IGFBP5 and STK4 (Table 2) were significantly up-regulated in MCF-7-Tet-ON-FAK cells in the presence of doxycycline and down-regulated in MCF-7-FAKsiRNA cells. In summary, FAKsiRNA decreased FAK expression and tumorigenesis *in vivo*, and Affymetrix gene expression analysis revealed more than 4300 genes that are up- and down-regulated by FAK siRNA, that are critical for studying molecular mechanisms of down-stream FAK signaling during breast tumorigenesis *in vivo*.

## Discussion

FAK controls survival and is activated at the early stages of breast carcinogenesis [25]. FAK expression was demonstrated in ductal carcinoma in situ (DCIS) tumors. The study suggested that FAK overexpression occurred in preinvasive, DCIS tumors preceding tumor metastasis. In addition, we have shown that FAK formed a protein complex with vascular endothelial receptor-3 protein, VEGFR-3 in breast cancer cells [33] suggesting its critical role in breast lymphogenesis and angiogenesis.

Thus, in the present study we performed analysis of induced FAK expression and induced dominant-negative FAK, FAK-CD in breast carcinogenesis using MCF-7-Tet-ON model. We generated MCF-7-tet-inducible cells, stably transfected with Tet-inducible FAK or Tet-inducible FAK-CD plasmids and performed analysis of this induced expression on the cellular processes *in vitro *and tumorigenesis *in vivo*. Increased expression of FAK increased cell growth, adhesion and soft agar colony formation *in vitro*. In contrast, induced expression of FAK-CD decreased these cellular processes. We inoculated these cells in nude mice and demonstrated increased tumorigenesis in the case of induced FAK and reverse processes in the presence of induced FAK-CD. Expression of FAK-CD caused cell rounding, which is explained by exogenous localization of FAK-CD in the focal adhesion and displacement of FAK from the focal adhesion sites [20]. The data are consistent with our previous report, where we have shown that exogenous FAK-CD can inhibit FAK functions and cause cell rounding and apoptosis of BT474 breast cancer cells [34]. FAK-CD or FRNK has been shown to decreased cell motility of AU-565 breast cancer cells [35]. In this report, we for the first time analyzed the direct effect of FAK and FAK-CD Tet-inducible expression on gene expression and cellular processes in MCF-7 line and in breast xenograft models. The increased tumorigenesis was accompanied by increased FAK mRNA and protein levels. Real-time PCR analysis demonstrated specific increased FAK mRNA in MCF-7-Tet-ON-FAK cells. Importantly, the expression of homologous Pyk-2 was not increased and was even decreased in MCF-7-Tet-ON-FAK cells indicating FAK-independent regulation of Pyk-2 in MCF-7 cells. Although, the recent report demonstrated increased expression of Pyk-2 and FAK in tissues from early and advanced breast cancers suggesting importance of Pyk-2 pathway in breast tumorigenesis [10], the down-stream signaling mediated by FAK and Pyk-2 kinases is different. The functional differences between Pyk-2 and FAK kinases are supported by the recent report on the structural differences between C-terminal FAT domains of FAK and Pyk-2 and differences in association and phosphorylation of focal adhesion protein, paxillin [36]. In this study we show that silencing of FAK with two different FAKsiRNA in MCF-7 stable cell line resulted in decreased breast tumorigenesis *in vivo *and decreased FAK expression in the tumor samples. We revealed significant differences in gene expression affected by FAK silencing or FAK up-regulation in MCF-7 cells. Thus, FAK is critical for breast cell survival and tumorigenesis. The models can be used for targeted therapy and for studies of FAK inhibitors.

Intriguingly, MCF-7-Tet-ON-FAK cell line and tumors grown in the presence of doxycycline had decreased DMP1 (cyclin D binding protein 1) mRNA and protein levels. DMP1 is a transcription factor which binds to cyclin D and when overexpressed induces cell cycle arrest [37]. DMP1 can bind Arf1 (p14^Arf ^known as an inhibitor of Mdm-2 and stabilizer of p53) promoter and activate its transcription, thus regulating Arf-p53 pathway. It is known that loss of DMP1 caused spontaneous tumorigenesis in mice and death by 24 months of age from different forms of cancer [38]. Dmp1^-^/^- ^mice among phenotypic abnormalities had also poor mammary development [37]. The Dmp1^+^/^- ^tumors often retain wild type allele of DMP1, thus DMP1 is haplo-insufficient for tumor suppression [37]. Overexpression of cyclin D1, which is found to be overexpressed in 60–80% of breast cancer tumors, inhibits the transcriptional activity of DMP1 and antagonizes its function [37]. It was shown that overexpression of FAK increased expression of cyclin D1 [39], which contributed to increased expression of cellular proliferation. The function of human DMP1 protein (the transcription factor that is involved in the oncogene-tumor suppressor signaling) is an unexplored area in human cancer, and it remains to discover its post-translational modifications and identification of DMP1-protein binding partners [37]. Thus, FAK-DMP1-cyclin D1 linked pathway can be a novel mechanism regulating intracellular functions and carcinogenesis, and decreased DMP1 expression can explain the increased cellular growth and tumorigenesis of the MCF-7-Tet-ON-FAK model.

Similar to adenoviral expression of FAK-CD that caused increased apoptosis [20,28], Tet-inducible MCF-7-Tet-ON-FAK-CD cells showed decreased viability and growth on soft agar. Interestingly, TaqMan analysis demonstrated significantly decreased AKT level in MCF-7-Tet-ON-FAK-CD tumors. These data are consistent with our previous report, when adenoviral FAK-CD decreased AKT in breast cancer cell lines [28]. We have shown that AKT increased survival of the breast cancer cell line [28]. Thus, down-regulation of AKT by doxycycline-induced FAK-CD can explain decreased tumorigenesis in these tumors.

We demonstrated that silencing of FAK with two FAK siRNA decreased tumorigenesis in MCF-7 xenograft model that was accompanied by decreased FAK expression in tumor samples. Importantly, we performed Affymetrix chip microarray analysis and for the first time demonstrated more than 4300 genes significantly up- and down-regulated, and >160 genes that are >2-fold down or up-regulated (p < 0.05) in FAKsiRNA#1 and #2 samples group compared to control group that provides basis for future mechanistic detail study of FAKsiRNA-directed gene expression in breast cancer cells. The most important that both microarray data and TaqMan Real-time PCR array data demonstrate significantly decreased expression of FAK in FAKsiRNA cell lines compared to the control group, and these data together with decreased tumorigenesis *in vivo *support the critical role of FAK signaling in breast tumorigenesis.

The data on 169 genes that were 2-fold significantly up or down-regulated by FAKsiRNA in MCF-7 breast cancer model provide a basis for detail mechanistic study of FAK down-stream signaling during breast tumorigenesis. Several genes that were up-regulated by FAKsiRNA, like MAP2K5, mitogen-activated protein are connected with the FAK signaling pathway. We have shown that FAK up-regulated ERK1/2 in the stress conditions [28] in breast cancer cells. MAP2K5 (MEK5) kinase has been shown to be correlated with metastasis in prostate tumors [40] and has been involved in breast carcinogenesis[41]. Other important genes down-regulated by both FAKsiRNA#1 and #2 was PEG10, paternally expressed 10 gene that is directly involved in tumorigenesis [42]. PEG10 is a c-Myc target gene in cancer cells and has been shown to be activated during breast tumorigenesis, expressed in 55% of ductal carcinoma *in situ *and 32% of invasive ductal carcinoma [43]. Thus, down-regulation of PEG10 by FAKsiRNA is consistent with decreased tumorigenesis in these cells. Another important gene that was down-regulated by FAKsiRNA is PSAT1, phosphoserine aminotransferase. Recently, it has been shown that PSAT1 overexpression stimutated cell growth of colon cancer cells [44]. High PSAT1 mRNA levels were associated in breast cancers with poor clinical response to endocrine therapy [45]. Thus, PSAT1 functions as pro-survival and proliferative factor in tumorigenesis.

Among up-regulated genes (>3-fold) by FAKsiRNA was thiredoxin interacting protein (TXNIP) that is known to be involved in apoptosis [46]. In pancreatic Miapaca-2 cells, overexpression of TXNIP resulted in increased basal apoptosis and increased sensitivity to cisplatin and oxaliplatin [46]. We also performed Affymetrix array analysis on Tet-ON-FAK MCF-7 cells and found that thiredoxin interacting protein (TXNIP) was 2.7 fold decreased in the presence of doxycyline, supporting that TXNIP is regulated by FAK and plays role in breast tumorigenesis. We revealed several genes that were inversely regulated in MCF-Tet-ON-FAK with induced FAK and in MCF-7 cells with silenced RNA, 16 genes were >1.5 fold in inversely regulated, p < 0.05 (not shown), providing a basis for the mechanism of their regulation. Another gene that was ~5 fold up-regulated by FAKsiRNA is transcription factor AP-2 alpha (TFAP2A). Activation and expression of AP-2 was associated with increased apoptosis and inhibiton of cell growth [47]. In addition, immunohistochemical staining studies showed that loss of transcription factor AP-2 correlated with disease progression from normal breast to invasive breast cancer disease [48]. Another group demonstrated that reduced expression of AP-2 transcription factor associated with aggressive breast cancer [49]. Down-regulation of AP-2 with siRNA led to enhanced breast cancer tumor growth and reduced chemotherapy-induced cell death [50]. Thus, these few examples of down-regulated and up-regulated genes can explain reduced tumorigenesis in FAKsiRNA MCF-7 model and suggest that FAKsiRNA can be used as a therapy approach.

The presented Tet-regulated FAK-CD, dominant negative FAK, breast cancer cell model can be compared with Tet-inducible dominant-negative c-Src model [51]. Similar to dominant-negative c-Src-induced model of Tet-ON MCF-7 cells, cells with induced FAK-CD had decreased cell adhesion and viability and reduced tumorigenesis, consistent with our data on cooperative survival signaling of FAK and Src in colon cancer cells [22].

## Conclusion

In conclusion, we have shown for the first time that Tet-inducible FAK increased cell growth, adhesion and soft agar colony formation *in vitro*, while it increased breast tumorigenesis *in vivo*. In contrast, Tet-inducible FAK-CD and FAKsiRNA blocked these processes *in vivo*. TaqMan Low Density Array identified several specific genes affected by modulation of FAK expression and function, such as DMP1 and AKT1. In addition, Affymetrix analysis revealed more than 4300 genes that were affected by FAKsiRNA in MCF-7 xenograft tumors. This system demonstrates the basis for future studies on the role of FAK and downstream signaling in breast tumorigenesis and the biology of breast cancer cells. Thus, FAK is a promising target for future breast cancer therapy.

## List of abbreviations

FAK: Focal Adhesion Kinase; FAK-CD: C-terminal domain of FAK; siRNA: small interfering RNA; DSIC: ductal carcinoma in situ; DMP1: cyclin D binding protein 1; Tet: tetracycline; Dox: doxycycline.

## Competing interests

The authors declare that they have no competing interests.

## Authors' contributions

WGC supervised the study. VMG planned the experiments and supervised the experimental work. MZ conducted experiments with generation cell lines and mice experiments. LZ performed Real-time PCR analyses. J-Li performed microarray and statistical analyses. All authors approved the manuscript.

## Pre-publication history

The pre-publication history for this paper can be accessed here:

http://www.biomedcentral.com/1471-2407/9/280/prepub

## Supplementary Material

Additional file 1 **The Taq Man Low Density Array gene set**. The data presented in a Table show the Taq Man Low Density Array gene set used for Real-time PCR analysis. Click here for file
