# Supplementary material for: The direct effect of Focal Adhesion Kinase (FAK), dominant-negative FAK, FAK-CD and FAK siRNA on gene expression and human MCF-7 breast cancer cell tumorigenesis
Source: BMC Cancer. 2009 Aug 12;9:280. doi: 10.1186/1471-2407-9-280 (PMC3087335; doi:10.1186/1471-2407-9-280)
Supplement: Additional file 1 — The Taq Man Low Density Array gene set. The data presented in a Table show the Taq Man Low Density Array gene set used for Real-time PCR analysis. [file 1471-2407-9-280-S1.doc]

**The TaqMan Low Density Array gene set**

| **Gene Symbol** | **Gene Name** | **Sequence reference** | **Gene Aliases** |
| --- | --- | --- | --- |
| PTK2 | PTK protein tyrosine kinase 2 | NM_153831.2 | FADK; FAK; FAK1; pp125FAK |
| PTK2B | PTK2B protein tyrosine kinase 2 beta | NM_173174.1 | CADTK; CAKB; FADK2; FAK2;  PYK2; PKB;RAFTK |
| TP53 | tumor protein p53 (Li-Fraumeni syndrome) | NM_000546.3 | LFS1, TRP53, p53 |
| DMTF1 | cyclin D binding myb-like transcription factor 1 | NM_021145.2 | DMP1;DMTF; FLJ41265; hDMP1 |
| CASP3 | caspase 3, apoptosis-related cysteine peptidase | NM_032991.2 | CPP32;CPP32B:SCA-1 |
| SRC | v-src sarcoma (Schmidt-Ruppin A-2) viral oncogene | NM_198291.1 | ASV; SRC1, c-SRC, p60-Src |
| MAPK3 | mitogen-activated protein kinase 3 | NM_002746.2 | ERK-1; ERT2; P44MAPK |
| AKT1 | v-akt murine thymoma viral oncogene homolog 1 | NM_005163.2 | AKT;MGC99656;PKB;PRKBA;  RAC;RAC-ALPHA |
| MAP2K1 | mitogen-activated protein kinase kinase 1 | NM_002755.2 | MAPKK1;MEK1;MKK1;PRKMK1 |
| MAPK8 | mitogen-activated protein kinase 8 | NM_139047.1 | JNK;JNK1;JNK1A2;JNK21B1/2;  PRKM8;SAPK1 |
| GAPDH | glyceraldehyde-3-phosphate dehydrogenase | NM_002046.3 | G3PD;GAPD;MGC88685 |
| FYN | FYN oncogene related to SRC, FGR, YES | NM_002037.3 | MGC45350;RP1-66H14.1;SLK;SYN |
| CDC2 | cell division cycle 2, G1 to S and G2 to M | NM_033379.2 | CDC28A;CDK1;DKFZp686L20222 |
| CDK4 | cyclin-dependent kinase 4 | NM_000075.2 | CMM3;MGC14458;PSK-J3 |
| RB1 | retinoblastoma 1 (including osteosarcoma) | NM_000321.2 | OSRC;RB |
| SOCS2 | suppressor of cytokine signaling 2 | NM_003877.3 | CIS2;Cish2;SOCS-2;SSI-2;SSI2;STATI2 |
| TIE1 | tyrosine kinase with immunoglobulin-like and EGF-like | NM_005424.2 | JTK14;RP11-282K6.6;TIE |
| SYK | spleen tyrosine kinase | NM_003177.3 | DKFZp313N1010; EC 2.7.10.2; FLJ25043 |
| PDGFRB | platelet-derived growth factor receptor, beta polypeptide | NM_002609.3 | CD140B;JTK12;PDGF-R-beta; PDGFR; DGFR1 |
| CDK2 | cyclin-dependent kinase 2 | NM_001798.2 | p33(CDK2) |
| CDK3 | cyclin-dependent kinase 3 | NM_001258.1 | EC 2.7.11.2 |
| RAF1 | v-raf-1 murine leukemia viral oncogene homolog 1 | NM_003215.2 | CRAF;Raf-1;c-Raf |
| ABL-1 | v-abl Abelson murine leukemia viral oncogene homolog 1 | NM_007313.2 | ABL;JTK7;RP11-83J21.1;bcr/abl;  c-ABL;p150;v-abl |
| TEC | tec protein tyrosine kinase | D29767.1 | MGC126760,MGC126762;PSCTK4 |
| PXN | Paxillin | NM_002859.1 | FLJ16691;hCG_1778014 |
| SHC1 | SHC (Src homology 2 domain containing) transforming | NM_003029.3 | FLJ26504;SHC;SHCA;p52SHC;p66;  p66SHC |
| BCAR1 | breast cancer anti-estrogen resistance 1 | NM_014567.2 | CAS;CRKAS;P130Cas |
| MAP2K6 | mitogen-activated protein kinase kinase 6 | NM_002758.3 | MAPKK6;MEK6;MKK6;PRKMK6;  SAPKK3 |
| EPHA1 | EPH receptor A1 | NM_005232.3 | EPH;EPHT;EPHT1;MGC163163 |
| CTNNB1 | catenin (cadherin-associated protein), beta 1, 88kDa | NM_001904.3 | CTNNB;DKFZp686D02253;FLJ25606; |
| CHEK1 | CHK1 checkpoint homolog (S. pombe) | NM_001274.3 | CHK1 |
| ATM | ataxia telangiectasia mutated | NM_000051.3 | AT1;ATA;ATC;ATD;ATDC;ATE;  TEL1;TELO1 |
| BIRC5 | baculoviral IAP repeat-containing 5 (survivin) | NM_00168.2 | API4;EPR-1 |
| CDC25C | cell division cycle 25 homolog C (S. pombe) | NM_001790.3 | CDC25 |
| BCL2 | B-cell CLL/lymphoma 2 | NM_000633.2 | Bcl-2 |
| TLN2 | talin 2 | NM_015059.1 | DKFZp451B1011;DKFZp686I0976;  KIAA0320 |
| FLT1 | fms-related tyrosine kinase 1; vascular endothelial growth factor receptor 1 | NM_002019.3 | FLT;VEGFR1 |
| ESR2 | estrogen receptor 2 (ER beta) | NM_001437.2 | ER-BETA;ESR-BETA;ESRB;ESTRB;Erb; |
| PINK1 | PTEN induced putative kinase 1 | NM_032409.2 | BRPK;FLJ27236;PARK6 |
| STAT1 | signal transducer and activator of transcription 1, 91kDa | NM_139.266.1 | DKFZp686B04100;ISGF-3;STAT91 |
| ARHGEF2 | rho/rac guanine nucleotide exchange factor (GEF) 2 | NM_004723.2 | DKFZp547L106;DKFZp547P1516;  CFH1;KIAA0651;LFP40;P40 |
| ITGB1 | integrin, beta 1 | NM_133376.1 | CD29;FNRB;GPIIA;MDF2;MSK12;VLAB |
| CELSR1 | cadherin, EGF LAG seven-pass G-type receptor 1 | NM_014246.1 | CDHF9;DKFZp434P0729;  FMI2;HFMI2; ME2 |
| RASGRP3 | RAS guanyl releasing protein 3 (calcium and DAG-regulated) | NM_170672.1 | GRP3;KIAA0846 |
| LAMC2 | laminin, gamma 2 | NM_018891.1 | B2T;BM600;EBR2;EBR2A;  LAMB2T;LAMNB2; |
|  |  |  |  |
